# Experimental Infections of Wild Birds with West Nile Virus

**DOI:** 10.3390/v6020752

**Published:** 2014-02-13

**Authors:** Elisa Pérez-Ramírez, Francisco Llorente, Miguel Ángel Jiménez-Clavero

**Affiliations:** Centro de Investigación en Sanidad Animal (CISA), Instituto Nacional de Investigación y Tecnología Agraria y Alimentaria (INIA), Valdeolmos (Madrid), 28130, Spain; E-Mails: dgracia@inia.es (F.L.); majimenez@inia.es (M.A.J.-C.)

**Keywords:** West Nile virus, wild birds, experimental infection, pathogenesis, transmission, immunity, host competence

## Abstract

Avian models of West Nile virus (WNV) disease have become pivotal in the study of infection pathogenesis and transmission, despite the intrinsic constraints that represents this type of experimental research that needs to be conducted in biosecurity level 3 (BSL3) facilities. This review summarizes the main achievements of WNV experimental research carried out in wild birds, highlighting advantages and limitations of this model. Viral and host factors that determine the infection outcome are analyzed in detail, as well as recent discoveries about avian immunity, viral transmission, and persistence achieved through experimental research. Studies of laboratory infections in the natural host will help to understand variations in susceptibility and reservoir competence among bird species, as well as in the epidemiological patterns found in different affected areas.

## 1. Introduction

West Nile virus (WNV, *Flaviviridae*, *Flavivirus*) is an emerging zoonotic arbovirus (arthropod-borne virus) widely distributed throughout the world and with considerable impact both on public health and on animal health [1]. It was first isolated in 1937 from the blood of a febrile woman in the West Nile district of Uganda, hence the name of the virus [2]. WNV is maintained in nature in an enzootic cycle involving ornithophilic mosquitoes as transmission vectors and certain birds as reservoir hosts. Spillover from this cycle occasionally results in severe outbreaks. 

West Nile virus is an ecological generalist [3] with an extraordinarily complex eco-epidemiology. Many species of birds act as primary hosts for WNV, though its vertebrate host range includes also species of mammals, amphibians, and reptiles [4]. Not all infected hosts transmit the virus, but only those in which the virus replicates efficiently enough to reach viremias sufficiently high to infect mosquitoes through blood feeding. This is called “host competence” and is a characteristic of each host species in a specific host-virus-vector system. For instance, a viremia level of 10^4^–10^5^ pfu/mL has been established as necessary for infection of feeding *Culex* mosquitoes [5]. Competent hosts for WNV transmission are found almost exclusively among avian species [6]. Among arthropod vectors, WNV also replicates in a wide range of mosquitoes. For instance, it has been found infecting up to 59 different species of mosquitoes in the US [7] and up to 28 in Italy [8]. Other arthropods, like ticks, are also susceptible to WNV infection, and the virus has been repeatedly isolated from both soft and hard ticks [9], although their role as vectors of WNV transmission is still uncertain. As occurs with hosts, not all infected vectors transmit the virus efficiently, but just those in which the virus replicates systemically and reach enough virus levels in the salivary glands to enable transmission after biting the host. This is called “vector competence” and is a characteristic of each vector species in a specific virus-vector system. *Culex* mosquitoes (*Diptera: Culicidae*), and particularly ornithophilic species (e.g., *Culex pipiens*) play an important vector role. However, some non-Culex species are also competent vectors (e.g., *Aedes albopictus*), though their role in transmission is unclear. This complex eco-epidemiology, involving hundreds of different vectors and hosts, which differ between locations, has likely contributed to the broad geographical range of WNV that has increased notably in the recent years, leading to its current consideration as the most widespread mosquito-borne flavivirus [10]. The geographical distribution of WNV at present covers large territories in all the continents, except Antarctica. Numerous episodes of WNV emergence have occurred in recent years in America and Europe [1], making WNV one of the best examples of emerging/re-emerging pathogens one can put forward.

West Nile virus is known to cause a severe, life-threatening neurological disease in humans and horses. However, the majority of WNV infections are actually asymptomatic. In humans, approximately 20% of the infected individuals develop a self-limiting febrile illness called “West Nile fever” (WNF), a flu-like clinical condition lasting about four to six days, characterized by high fever, malaise, headache, eye and muscle pain, nausea, vomiting, diarrhea, and other mild clinical signs. A lower proportion (approximately 1 in every 140 infected individuals) develop a severe affection of the central nervous system (CNS), called “West Nile neurological disease” (WNND), a neuroinvasive disease characterized by meningitis, encephalitis, paresis or paralysis, which is lethal in 4% to 14% of the neurological cases. The risk to develop severe disease increases with age [11].

In addition, in horses, the majority of the infections are asymptomatic. About 10% of clinically affected horses suffer from a severe neuroinvasive disease characterized by encephalitis and neurological signs (paralysis of the limbs, facial tremors), which is lethal in approximately one third of the cases [12].

Apart from horses and humans, other vertebrate hosts susceptible to WNV disease include a range of reptiles, mammals and birds [4]. Among birds, though the virus was shown to be pathogenic for crows and other wild bird species in early works [13], relevant wild bird mortalities in the field have only been observed in Israel since 1998 [14] and in North America since its first observation in 1999 [15].

As noted above, recent years have witnessed an outstanding expansion of WNV along with a rise in WN disease incidence [16]. In parallel, significant changes have been observed in its epidemiology and virulence. In the past, WNV was considered a pathogen of lesser importance, causing a mild disease in humans, during sporadic, small, self-limiting outbreaks resolving spontaneously. This notion has changed as WNV is causing large, persistent epidemics, mainly in North America since its first occurrence there in 1999. Particularly noteworthy is the increase in pathogenicity for wild birds, the amplifying hosts, which once were considered non-susceptible to the disease, and now suffer high mortalities in some instances. In Europe also, significant changes have occurred in its recent re‑emergence, leading to increasing outbreak frequency and persistence, human affection, and virulence for certain wild birds [1].

Free ranging bird mortalities are the hallmark of WN disease emergence in North America, while in the Old World wild bird mortality events are very infrequent, with small, sporadic episodes affecting one or few individuals, often detected in wildlife rehabilitation centres [17]. The little wild bird affection caused by WNV in the Old World as compared to North America, demands a satisfactory explanation, which is still pending. Hypotheses, such as different pathogenicity of the viruses circulating in each area and different susceptibility of local birds to WN disease have been proposed. However, given the wide diversity of WNV strains and lineages that are currently circulating in Europe and the peculiar eco-epidemiology of the virus in this continent, this issue will only be satisfactorily tackled by experimental studies comparing the course of the infection in Palearctic *vs.* Nearctic wild bird species inoculated with different WNV strains.

Likewise, several gaps in our knowledge, concerning pathogenesis, transmission routes and virulence of diverse WNV strains, can only be addressed through experimental infections in animal models. Rodents, and especially mice, are the most widely used *in vivo* experimental model of WNV disease in mammals. Researchers can capitalize on the availability of many different genetically and immunologically defined mouse strains to model flavivirus neurological disease, examine particular aspects of WNV virulence (such as neuropathogenicity and neuroinvasiveness), or discover host-pathogen interactions that influence disease outcome in humans and other mammals [18].

However, the most desired *in vivo* model to unravel the eco-epidemiology of WNV is the one closest to the natural reservoir, namely wild birds. Experimental infections of birds have been pivotal to assess the potential of different species to serve as amplifying hosts for the virus, to characterize the pathogenesis of infection with different strains and to evaluate the efficacy of vaccines and therapeutic agents [19]. Knowledge gained from these experimental trials has allowed to confirm field observations about species susceptibility, as well as to determine the role of local bird species in transmission, amplification, persistence and geographical dissemination of specific WNV strains. 

Although modeling WNV disease in the natural host affords several advantages, as aforementioned, limitations exist in relation to this experimental model. Contrary to other laboratory animals, such as mice or hamsters, in birds, the utilization of large numbers of animals is not always possible, especially if the target species is not farm bred, as is the case for most wild birds. Besides, collection of birds from the wild for scientific purposes is restricted and always implies Government collecting and possession permits. Wild origin of birds implies an important heterogeneity among collected birds in relation to age, sex, physical condition, or previous pathogen exposure. Moreover, classification and selection of individuals for experimental trials is not always possible and, thus, researchers must take host variability into account when interpreting results. As regards husbandry in BSL3 facilities, it is necessary to carefully consider that wild birds require expert handling, special housing facilities and time limited procedures to minimize stress derived from captivity, assuring, thereby, animal welfare, and avoiding biasing effects of stress on experimental infection results.

The objective of this review is to summarize the main achievements of WNV experimental research carried out in wild birds, highlighting advantages and limitations of this model. Viral and host factors that determine the infection outcome in wild birds will be analyzed in detail, as well as recent discoveries about avian immunity, viral transmission, and persistence.

## 2. Viral Factors

### 2.1. Lineages and Strains

As for other RNA viruses lacking proofreading replication, WNV genome is highly variable and consequently of extraordinary adaptability. As a result, many WNV variants have evolved independently in different parts of the world. As the virus moves from one area to another, either by nature, through migration and short distance movements of vertebrate hosts and/or invertebrate vectors or by human influence (trade and/or other activities), WNV strains from different origins can coexist (and co-evolve) in a particular area. This is the case in Europe, where several introduction events have been documented and at least five WNV genetic lineages have been identified to date [20]. This situation is clearly different from that of North America, where the circulating lineage 1 originated from a unique introduction that occurred in 1999. As a result, WNV strains circulating in each region might differ in their biological properties, and particularly in their pathogenicity. Consequently, characterization of strain virulence, as well as other phenotypic traits, is of paramount importance for a better understanding of WNV occurrence in a given area, notably when different lineages/strains/variants co-circulate in the same geographical region.

Experimental infections in wild birds were first carried out with viruses of lineage 1a (Egypt strain) [13] and lineage 2 (South African strain) [21,22]. While the South African isolate caused no specific illness or mortality in a wide variety of wild birds [21], the Egyptian strain (An248) caused disease in Passeriformes (Hooded crow (*Corvus cornix*) and House sparrow (*Passer domesticus*)) but not in other orders (Laughing dove (*Spilopelia senegalensis*), Common kestrel (*Falco tinnunculus*) or Cattle egret (*Bubulcus ibis*)). However, the deaths of at least some of the birds were attributed to the stress of handling and captivity [4]. Later on, Boyle *et al.* [23,24] performed experimental infections of herons in Australia using lineage 1b (Kunjin strain). Again, viremia was low and no mortality was associated with the infection. The results of these early experimental studies, together with the absence of WNV associated mortality in the field led to the idea that circulating strains at that time were not pathogenic for birds, causing only subclinical disease with varying levels of viremia [4].

With the introduction of WNV in the US in 1999, a striking change in WNV epidemiology occurred. This year, the virus appeared in New York City presaging the largest WNV epidemic in history in 2003, with thousands of human cases of WNND and large numbers of crows and other wild birds dying from the infection throughout temperate North America. Thereafter, the number of experimental studies using the American prototype strain NY99 greatly increased [5,25,26] in an attempt to understand the huge impact of the disease in America as compared to what had been previously observed in the Old World.

To date, two thirds of experimental studies carried out in wild birds have been performed with the NY99 strain (Table 1), biasing current knowledge towards only one of the existing lineages (lineage 1a), which may have important consequences influencing available data about host and vector competence, clinical patterns and diagnostic methods, among others. After the first occurrence of WNV in the US, the virus continued to spread and evolve relentlessly throughout North, Central and South America, giving rise to new isolates and variants belonging to the same lineage 1a. Of those, several have been recently assayed in experimental studies using wild birds as models. This is the case for isolates from Texas and Mexico [27], California [28], Colorado [29], and Argentina [30].

Although the actual mechanism or date of WNV introduction in America probably will never be known, it seems likely that the virus was introduced by air traffic from the Middle East, linked to the outbreaks reported in Israel in 1998 [31], caused by a lineage 1 strain closely related to the one that invaded US the following year. This strain affected not only humans, but also domestic geese and was also responsible for the death of White storks (*Ciconia ciconia*) in Southern Israel during their migration to Africa from central Europe [32]. Two experimental infections carried out with the Israel 1998 strain have demonstrated high virulence in Common goose chicks (*Anser anser*) [33] and Carrion crow (*Corvus corone*) [34]. In both species, high viral loads in organs and feathers were detected after infection, which might contribute to WNV direct transmission through cannibalism and feather-picking [33,34].

Among African strains, apart from the ones aforementioned (Egypt and South Africa), only two others, both belonging to lineage 1, have been studied in experimental infections: Kenya 3892 [26,35,36] and Morocco 2003 [37]. Both isolates caused medium to high viremia and mortality rates depending on the infected avian host.

Studies addressing specifically the effect of European WNV strains in wild birds have only been started recently, most of them focusing on lineage 1 isolates. Two of them inoculated Western‑Mediterranean WNV strains, namely, France 2000 and Spain 2007 [34,37] in Carrion crow and Red-legged partridge (*Alectoris rufa*), respectively. These studies showed that both strains are pathogenic for the assayed Palearctic wild bird species, though, in Carrion crow, the strain France 2000 caused less mortality and less viremia than the strain Israel 1998. Another study showed that different Euro-Mediterranean WNV strains, including Spain 2007 and Italy 2009, are pathogenic for House sparrow, but with reduced capacity for replication in—and transmission from—this host, as compared to NY99 strain, a feature that could help to understand the lower incidence of the disease in birds observed in Europe in comparison with North America [38]. 

Before 2004, WNV lineage 2 was considered to be restricted to sub-Saharan Africa. However, in 2004, lineage 2 strains were almost simultaneously detected in Hungary and Southern Russia [39,40,41]. The Hungarian strain, that was isolated from diseased goshawks (*Accipiter gentilis*) [40], continued to spread from central Europe to other countries and has been detected in Greece, Austria, and Italy, in 2011 causing disease in humans, birds and horses [39]. Meanwhile, the Russian strain also spread through a wide geographical area in Russia causing human disease and was detected in Romania in 2010 [42]. Until now, only one experimental study has been published in which the course of infection of a European lineage 2 strain (Austria 2009) is compared to that caused by a lineage 1 strain (NY99) in a wild bird species [43]. In this study, the authors have confirmed that both lineages can cause high mortality rates (33%) and that the assayed species (Gyrfalcon, *Falco rusticolus*) can act as a competent host for both strains, reaching similar viremia levels clearly above the established threshold of infectious viremia for *Culex pipiens* mosquitoes.

### 2.2. Pathogenicity Determinants

Micro-evolutionary changes, observed naturally due to adaptation to local transmission cycles as the virus circulates and spreads, can generate new genotypes potentially associated to phenotypic changes altering virulence, neuro-invasiveness, transmissibility and vector and host range [27]. As a result, WNV strains circulating in each region might differ in their biological properties, and particularly in their pathogenicity. Consequently, characterization of strain virulence and pathogenicity determinants is of paramount importance for a better understanding of WNV occurrence in a given area.

For this purpose, mice have been the most widely used *in vivo* model. Through the integration of information on neuropathogenicity and neurotropism in mice with the analysis of genetic changes occurring in field WNV isolates and reverse genetics (using infectious clones, molecular chimeras, or other strategies), relevant information on virulence determinants has been gathered [44,45,46]. Genetic analyses and inoculation of mice with viruses generated through site-specific mutagenesis have revealed that pathogenicity determinants can occur in both structural (E) [44] and non-structural (NS2A, NS3, NS4B) [35] genes and that strain virulence can also be influenced by changes at both 5' and 3' non-coding regions [46,47]. However, only few of the identified determinants of viral virulence in mice have been confirmed in natural avian hosts. 

Glycosylation of the virion envelope glycoprotein (E) of WNV has been reported to be responsible for the increased pathogenicity in mice [48,49]. This result has also been confirmed in birds, both using *in vitro* (avian cells) and *in vivo* systems (two-days old chicks) [50,51]. Furthermore, Brault *et al.*, [27] have confirmed that a WNV variant from Mexico that presented the E-glycosylation motif produced higher viremias and shorter survival times in American crow (*Corvus brachyrhynchos*) and House sparrow than variants that lacked of this E-glycosylation [27]. In this study, strains that were non-neuroinvasive in mice still caused significant mortality in birds, suggesting that variable pathogenic mechanisms of virulence and attenuation are present in these vertebrate models and that the murine model may not accurately predict virulence in birds. 

Even among avian hosts, results of *in vivo* experiments using recombinant viruses in a given species cannot be blindly extended to other susceptible avian species. That is the case of the experiment carried out by Brault *et al.* [35] in which a single positively selected mutation at the NS3 gene (T_249_P amino acid substitution) was sufficient to generate a phenotype highly virulent for American crow. However, this effect could not be reproduced in House sparrow [36]. Therefore, specific mutations related to increased pathogenicity in a given species should not be assumed to be more pathogenic for any host without being assessed experimentally. In addition, Mediterranean WNV lineage 1a strains with the NS3_249_P genotype showed less pathogenicity than their NS3_249_T counterparts not only in mice [52] but also in a bird species indigenous to Southern Europe, the Red-legged partridge [37]. This suggests that a proline residue in position 249 of the NS3 protein is not sufficient to enhance virulence for any given WNV strain. In this study, however, it cannot be excluded that other residues that differ between both strains could also have had an influence in the observed pathogenicity. 

### 2.3. Viral Dose

Free-ranging birds are exposed to a variable range of WNV doses via mosquito bite and thus, assessment of host susceptibility is more complex that can be determined by inoculation of a single dose in experimental infection studies [53]. Knowledge of potential dose-dependent responses among a variety of avian species is relevant for understanding host reservoir potential and transmission dynamics [54]. However, this factor has been overlooked until recently, when several experimental studies have specifically investigated the response of avian hosts to various viral doses, administered both by mosquito bite [53,55] and by needle inoculation [43,54,56]. These studies have provided consistent results as regards the effect of viral dose on probability of infection, viremia levels and clinical outcome. In general terms, viremia titres and morbidity did not increase in a dose dependent manner. In most of the studied species, low viral doses were sufficient to overcome the host defense mechanisms and cause morbidity. In fact, in some cases, birds that succumbed to the infection had been inoculated with the lowest dose [43,54], indicating that disease outcome is a complex interplay of hosts, vectors, viral dose, and strain. Nonetheless, there was a clear correlation between the viral dose and the probability of becoming viremic after inoculation or mosquito bite, increasing in a dose dependent manner the proportion of inoculated birds that became viremic [54]. Likewise, higher doses resulted in more rapid onset of viremia and oral shedding [54,56], although there were no significant differences among doses after one to two days, when peak viremia titres were reached [53,54].

Apart from viral dose, other factors play important roles in WNV transmission. Some of them have been thoroughly studied in mice, such is the case of the enhancing effect of mosquito saliva [57,58], while others merit further research, as the effect of inoculation site, the viral source (invertebrate *vs.* vertebrate cells) or the potentiating effect of multiple mosquito bites [55].

## 3. Host Factors

### 3.1. Taxonomic Classification

**Figure 1:**
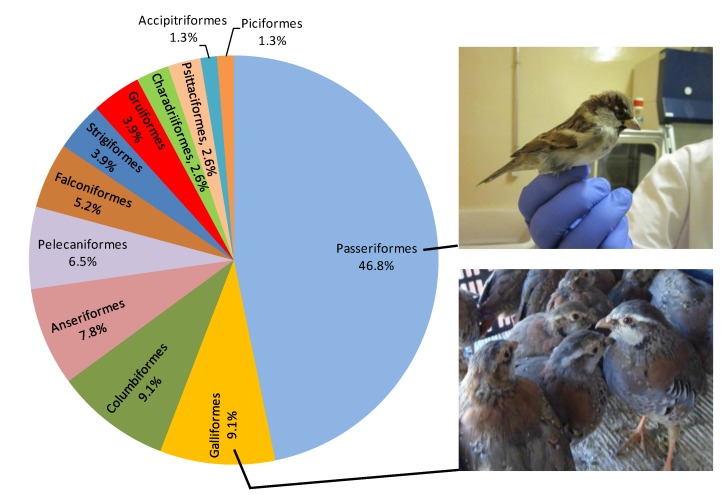
Percentage of wild bird species, distributed by order, used in WNV experimental infections. Pictures show two representative species used in this type of experiments by the authors’ group: House sparrow (*Passer domesticus*) and Red-legged partridge (*Alectoris rufa*).

**Figure 2:**
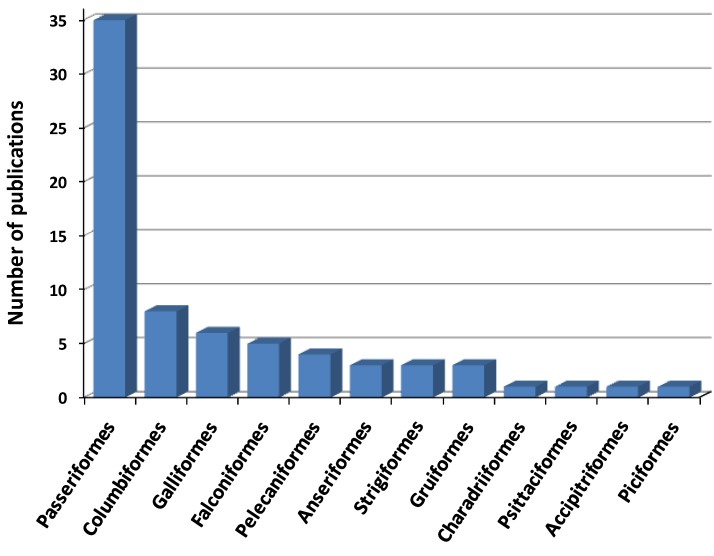
Number of publications involving WNV experimental inoculations in wild birds, distributed by taxonomic order.

A multitude of bird species have been evaluated by experimental infection in order to identify those that are competent hosts and to characterize response to infection (viremia levels, antibody production, viral shedding and clinical signs). As shown in Table 1 and Figure 1 and Figure 2, 77 wild bird species belonging to 29 families and 12 orders have been experimentally inoculated with different strains of WNV since 1955. In most cases, selection of species for experimental trials has been based on field observations of clinical disease and mortality, as is the case of Corvidae family whose high susceptibility to the virus has been confirmed through experimental infections [5,26,35,59].

One of the main goals of experimentally infecting wild birds with WNV is to accurately estimate their host competence, which is a function of the intensity and duration of viremia and the susceptibility of infection of the affected host [5,60]. Based on viremia levels and considering a threshold value of 10^4^–10^5^ pfu/mL of blood (that has been established as necessary for infection of feeding *Culex* mosquitoes) [5,61], avian hosts can be roughly classified as incompetent, moderately competent or highly competent for WNV transmission.

In general terms, Passeriformes (especially Corvidae, Fringillidae, and Passeridae families) and Charadriiformes (Laridae) are considered highly competent hosts, although differences in viremia levels have been evidenced depending on the species and the viral strain [5,21,62,63,64]. Birds that develop mean peak viremias of 10^4^–10^6^ pfu/mL can be considered moderately competent hosts, such as several species belonging to orders Anseriformes and Passeriformes. Finally, birds that sustain a viremic titre of less than 10^4^ pfu/mL are classified as incompetent hosts, as is the case of Columbiformes, Pelecaniformes, Psittaciformes, and Galliformes. However, data shown in Table 1 suggest that important variations in viremia levels exist at all taxonomic levels, even within the same family. For instance, in the Phasianidae family, Greater-sage grouse (*Centrocercus urophasianus*) and Red-legged partridge develop high viremia levels at least with the assayed strains. Another example is the American white pelican (*Pelecanus erythrorhynchos*), that has been found to be a moderate-high competent host for WNV [65], while the rest of species belonging to the Pelecaniformes order that have been experimentally inoculated developed very low viremia levels. For these reasons, extrapolations based on taxonomic relationships of birds must be made cautiously because they may lead to spurious conclusions.

It is important to remark that the aforementioned classification of species as highly or low competent WNV hosts is mostly based on results of experimental infections with the strain NY99. Therefore, it is plausible to argue that response to the infection (in terms of viremia levels) and, thus, classification as competent hosts, would differ when different strains or lineages are inoculated in the same species, as demonstrated by Brault *et al.* [26], Bingham *et al.* [59], Ziegler *et al.* [43], and Del Amo *et al.* [38], in American crow, Little raven (*Corvus mellori*), Gyrfalcon, and House sparrow, respectively.

Although 77 wild bird species have been studied by experimental infection, there are still numerous families of birds whose susceptibility and host competency remain unknown, especially in the Passeriformes order [64]. 

### 3.2. Geographical Origin (Paleartic *vs.* Neartic Species)

While the first experimental research carried out with WNV was focused mainly in African avian species [13,21], the introduction of the virus in North America in 1999 and its devastating effect in native species caused a shift in target species of experimental procedures. Thereupon, the huge majority of wild birds used as models of WNV have been American native species [5,53,66,67,68] (see Table 1). Only in recent years, have indigenous European birds been assayed as models of WNV infection [34,37]. Even though these studies are still limited, as regards number of avian species and viral strains tested, they have provided evidence that the studied European wild bird species can be clinically affected by WNV and that at least some Euro Mediterranean strains are pathogenic for Palearctic wild birds [34,37]. Thus, the apparently limited wild bird morbidity caused by WNV in Europe, as compared to the high virulence for WNV in wild birds in North America, demands an alternative explanation and warrants further experimental and field research.

### 3.3. Age

The effect of age in the susceptibility of WNV infection in wild birds has not been widely studied. Indeed, determining the relative competence of nestling, juvenile, and adult birds has been identified as a priority for research on WNV transmission [64]. It is well known that, in general terms, nestlings are more susceptible than adults to mosquito-borne viral infections [69,70], as nestling and young birds have minimal feather coverage [71] and lack of defensive behavior [72], which increases vulnerability to mosquito bites. Under natural conditions, the age of birds also seems to be an important factor in determining whether the virus causes disease and death, being chicks and juveniles much more susceptible, at least in some species, such as Common geese [73], American white pelican [74], Chukar partridge (*Alectoris chukar*), or Impeyan pheasant (*Lophophorus impeyanus*) [75]. These observations should be carefully considered when designing an experimental trial, so that the age of birds coincide with that of maximum susceptibility registered under field conditions. This was the case in the study by Sotelo *et al.* [37], in which the age of Red-legged partridges was selected based on epidemiological data from the outbreak of a closely related species, the Chukar partridge [75].

Nevertheless, few experimental studies have addressed specifically the age-related differences in susceptibility and viremia levels in wild birds. For example, Boyle *et al.* [23] and Nemeth *et al.* [76] have evidenced in various avian species increased duration or intensity of viremia in nestlings and juveniles, compared to adult birds, after infection with different lineages of WNV. In California quail (*Callipepla californica*) and Gambel’s quail (*Callipepla gambelii*), it was demonstrated that only chicks (two to three days old) sustained viremia levels high enough to be considered competent hosts, while quails of 13 weeks had lost this ability, developing very low viremia titres [68]. In this regard, an important gap exists in the reviewed literature: in 48% of studies, the age of infected birds was completely unknown. Only in 14.5% of the articles, the exact age of the birds used in the study was specified. In the rest (37.5%), the exact age was unknown but at least an indication was given, classifying the birds as adults (>1 year) or immatures (<1 year). This constraint is mostly related with the wild origin of birds. The exact age of individuals that have been captured from the wild is in most cases impossible to know and age-related differences in size or feather pattern only allows, in best cases, partial classification as nestlings, young, or adults. Results obtained in experimental infections of birds of undetermined age must be interpreted with caution, as important variations may exist both in host competence and clinical outcome between different ages.

### 3.4. Other Life History Traits

Examining disease in wild avian species with contrasting life histories or subjected to different immunosuppressive agents can provide insight into virulence of the pathogen and immunity of the host [77]. Stress has been found to exacerbate the outcome of viral infectious diseases in humans and animal models [78]. In the case of WNV infection, the aggravating effect of different stress paradigms (administration of glucocorticoids, acute exposition to cold, isolation, loud noise, *etc.*) has been thoroughly investigated in mice (reviewed in [19]). With reference to birds, Owen *et al.* [79] have recently examined the effect of stress on WNV morbidity and mortality in Northern cardinal (*Cardinalis cardinalis*) using corticosterone implants. Although no effect on body mass, viremia, or temperature was observed, mortality increased by 450%, which suggest that wild bird populations that inhabit in stressful environments may suffer higher mortality if exposed to WNV. In this respect, various authors have demonstrated that the stress of excessive handling associated to experimental infections (daily bleeding, swabbing, *etc.*) contributed significantly to the observed mortality of birds used in the study [80,81]. With these considerations in mind, maximum care must be taken in controlling biasing effects of stress, particularly in wild bird species that are highly susceptible to any kind of stress derived from captivity and frequent handling. Consequently, it is of paramount importance that the experimental design includes a control group of birds that will be sham-inoculated but subjected to the same housing, handling and sampling conditions. Only in this way can researchers control stress-related effects, avoiding misleading interpretation of infection results.

The effect of stress associated to migratory activity on infection outcome has also been assessed under experimental conditions in Swainson’s thrush (*Catharus ustulatus*) and Gray catbird (*Dumetella carolinensis*) [82]. In this case, artificially induced migratory status did not have an impact on viremia titres, as might be expected if individuals had been immunosuppressed during migration. Mortality did not increase either, as none of the infected birds died during the infection, regardless of the migratory status. However, four of the nine infected Swainson’s thrushes reduced their activity to non-migratory levels during the viremic period.

In some avian diseases, stress associated with mating, territoriality, migration, or simply seasonal changes in hormone levels can trigger relapses in chronically infected birds [83,84]. Although this possibility would have important epidemiological consequences as regards overwintering and consequent amplification of WNV and other flaviviruses, attempts to stimulate relapses of infection through experimental immunosuppresion in birds have failed so far [85,86].

Other life history traits, such as habitat preferences or mating/breeding systems, that affect the historic pathogen exposure pattern, could potentially influence susceptibility to WNV infection. To date, it has only been experimentally assayed in passerines by Reisen and Hahn [77]. In this study, the authors infected four species of taxonomically related blackbirds (Icteridae) that differ in geographic range, breeding behaviour and mating system. Brown-headed cowbird (*Molothrus ater*) was found to be innately more resistant to the infection, showing the lowest mean viremia, clearing the infection faster and developing lower antibody levels than the other blackbirds. The principal factor differentiating the Brown-headed cowbird from its relatives is its parasitic breeding system. This life history strategy incurs increased exposure to pathogens from their parenting species which could lead to the evolution of a robust immune system and enhanced disease resistance. The incompetent status of other brood parasite cowbird species has also been confirmed in a previous study [30].

Finally, the presence of preexisting antibodies also affects susceptibility to WNV infection and viremia levels [87] as will be discussed in detail below (subsection 5.2).

**Table 1:** Summary of experimental infections of West Nile virus performed in wild birds.

Order	Family	Species	Strain/Mortality^+^	Viremia	Distribution	Ref.
Passeriformes	Turdidae	American robin (*Turdus migratorius*)	**NY**	**H**	AM	[5,54]
		Swainson’s thrush (*Catharus ustulatus*)	**NY**	**M**	AM	[82]
		Clay-colored thrush (*Turdus grayi*)	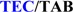	**M**	AM	[88]
	Corvidae	Carrion crow (*Corvus corone*)	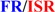	**L**	EUR/ASIA	[34]
		American crow (*Corvus brachyrhynchos*)	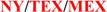	**H**	AM	[5,25,26,27,35,89,90,91,92]
			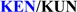	**M**		
		Fish crow (*Corvus ossifragus*)	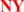	**H**	AM	[5,89,92]
		Little raven (*Corvus mellori*)	**NY**	**M**	OCE	[59]
			**KUN**	**L**		
		Hooded crow (*Corvus cornix*)	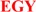	**H**	EUR/ASIA/AFR	[13]
		Western scrub-jay (*Aphelocoma californica*)	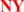	**H**	AM	[53]
		Blue jay (*Cyanocitta cristata*)	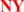	**H**	AM	[5,91]
		Black-billed magpie (*Pica hudsonia*)	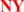	**H**	AM	[5]
		Jungle crow (*Corvus macrorhynchos*)	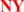	**H**	ASIA	[93]
	Passeridae	House sparrow (*Passer domesticus*)	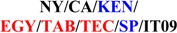	**H**	WORLDWIDE	[5,13,27,28,36,38,53,67,76,80,81,88,94]
			**TEX/KUN/IT08**	**M**		
			**MEX**	**L**		
		Cape sparrow (*Passer melanurus*)	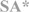	**L**	AFR	[21]
	Icteridae	Red-winged blackbird (*Agelaius phoeniceus*)	**NY**	**M/L**	AM	[5,77,95]
		Brown-headed cowbird (*Molothrus ater*)	**NY**	**L**	AM	[67,77]
		Brewer’s blackbird (*Euphagus cyanocephalus*)	**NY**	**H**	AM	[67,77]
		Tricolored blackbird (*Agelaius tricolor*)	**NY**	**H**	AM	[77]
		Common grackle (*Quiscalus quiscula*)	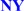	**H**	AM	[5]
		Great-tailed grackle (*Quiscalus mexicanus*)	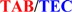	**H**	AM	[88]
		Bay-winged cowbird (*Agelaioides badius*)	**ARG**	**L**	AM	[30]
		Shiny cowbird (*Molothrus bonariensis*)	**ARG**	**L**	AM	[30]
	Emberizidae	Song sparrow (*Melospiza melodia*)	**NY**	**M**	AM	[96]
		White-crowned sparrow(*Zonotrichia leucophrys*)	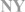	**na**	AM	[67]
	Fringillidae	Hawai’i ’amakihi (*Hemignathus virens*)	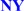	**H**	AM	[81]
		House finch (*Haemorhous mexicanus*)	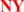	**H**	AM	[5,53,67,87]
Passeriformes	Ploceidae	African masked weaver (*Ploceus velatus*)	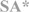	**M**	AFR	[21]
		Red-billed quelea (*Quelea quelea*)	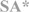	**L**	AFR	[21]
		Red bishop (*Euplectes orix*)	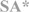	**M**	AFR	[21]
	Hirundinidae	Cliff swallow (*Petrochelidon pyrrhonota*)	**NY**	**M**	AM	[56,97]
	Mimidae	Gray catbird (*Dumetella carolinensis*)	**NY**	**M**	AM	[82]
		Northern mockingbird (*Mimuspolyglotto*s)	**NY**	**H**	AM	[94]
	Sturnidae	European starling (*Sturnus vulgaris*)	**NY**	**M**	WORLDWIDE	[5,67]
	Cardinalidae	Northern cardinal (*Cardinalis cardinalis*)	**NY**	**H**	AM	[79,94]
	Paridae	Tufted titmouse (*Baeolophus bicolor*)	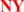	**H**	AM	[98]
	Troglodytidae	Carolina wren (*Thryothorus ludovicianus*)	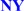	**H**	AM	[98]
Falconiformes	Falconidae	Gyrfalcon (*Falco rusticolus*)	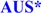	**H**	AM/EUR/AS	[43]
			**NY**	**M**		
		Hybrid falcon (*Falco rusticolus x Falco cherrug*)	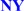	**L**	WORLDWIDE	[99]
		American kestrel (*Falco sparverius*)	**NY**	**H**	AM	[5,66]
		Common kestrel (*Falco tinnunculus*)	**EGY**	**L**	EUR/AS/AFR	[13]
Accipitriformes	Accipitridae	Red-tailed hawk (*Buteo jamaicensis*)	**NY**	**H**	AM	[66]
Strigiformes	Tytonidae	Barn owl (*Tyto alba*)	**NY**	**L**	WORLDWIDE	[66]
	Strigidae	Great horned owl (*Bubo virginianus*)	**NY**	**H**	AM	[5,66]
		Eastern screech-owl (*Megascops asio*)	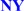	**H**	AM	[100]
Galliformes	Odontophoridae	California quail (*Callipepla californica*)	**NY**	**L**	AM	[53,68]
		Gambel’s quail (*Callipepla gambelii*)	**NY**	**L**	AM	[68]
		Northern bobwhite (*Colinus virginianus*)	**NY**	**L**	AM	[5]
	Phasianidae	Red-legged partridge (*Alectoris rufa*)	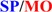	**H**	EUR	[37,101]
			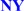	**L**		
		Japanese quail (*Coturnix japonica*)	**NY**	**L**	WORLDWIDE	[5]
		Ring-necked pheasant (*Phasianus colchicus*)	**NY**	**L**	WORLDWIDE	[5]
		Greater sage-grouse (*Centrocercus urophasianus*)	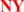	**M**	AM	[102]
Pelecaniformes	Ardeidae	Rufous night-heron (*Nycticorax caledonicus*)	**KUN**	**L**	OCE	[23,24]
		Little egret (*Egretta garzetta*)	**KUN**	**L**	EUR/AS/AFR/OCE	[23,24]
		Intermediate heron (*Mesophoyx intermedia*)	**KUN**	**L**	AFR/AS	[23,24]
		Cattle egret (*Bubulcus ibis*)	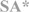 **/EGY**	**L**	WORLDWIDE	[13,21]
	Threskiornithidae	African sacred ibis (*Threskiornis aethiopicus*)	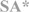	**L**	AFR/AS	[21]
Columbiformes	Columbidae	Rock pigeon (*Columba livia*)	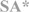 **/NY/TEC/TAB**	**L**	WORLDWIDE	[21,88]
		Ring-necked dove (*Streptopelia capicola*)	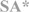	**L**	AFR	[21]
		Eurasian collared-dove (*Streptopelia decaocto*)	**NY/CO**	**M**	AM/EUR/AS/AFR	[29]
		Laughing dove (*Spilopelia senegalensis*)	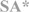 **/EGY**	**L**	AFR/AS	[13,21]
		Common ground-dove (*Columbina passerina*)	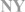	**na**	AM	[67]
		Mourning dove (*Zenaida macroura*)	**NY**	**M**	AM	[5,53,67]
		Picui ground-dove (*Columbina picui*)	**ARG**	**M**	AM	[30]
Gruiformes	Rallidae	American coot (*Fulica americana*)	**NY**	**L**	AM	[5]
		Crested coot (*Fulica cristata*)	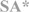	**L**	AFR/EUR	[21]
	Gruidae	Sandhill crane (*Grus canadensis*)	**NY**	**L**	AM	[103]
Anseriformes	Anatidae	Common goose (*Anser anser*)	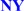	**M**	WORLDWIDE	[33]
		Canada goose (*Branta canadensis*)	**NY**	**M**	AM/EUR	[5]
		Mallard (*Anas platyrhynchos*)	**NY**	**H**	WORLDWIDE	[5]
		Yellow-billed duck(*Anas undulata*)	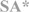	**L**	AFR	[21]
		Red-billed teal (*Anas erythrorhyncha*)	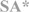	**L**	AFR	[21]
		Southern pochard (*Netta erythrophthalma*)	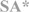	**L**	AFR	[21]
Charadriiformes	Charadriidae	Killdeer (*Charadrius vociferus*)	**NY**	**H**	AM	[5]
	Laridae	Ring-billed gull (*Larus delawarensis*)	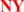	**H**	AM	[5]
Psittaciformes	Psittacidae	Monk parakeet (*Myiopsitta monachus*)	**NY**	**L**	AM	[5]
		Budgerigar (*Melopsittacus undulatus*)	**NY**	**L**	OCE	[5]
Piciformes	Picidae	Northern flicker (*Colaptes auratus*)	**NY**	**M**	AM	[5]

**CA**: California 04; **NY**: New York 99; **CO**: Colorado 08; **SA**: South Africa; **ARG**: Argentina 06; **EGY**: Egypt; **KUN**: Kunjin; **SP**: Spain 07; **MO**: Morocco 03; **AUS**: Austria 09; **MEX**: Mexico 03; **TEX**: Texas 03; **KEN**: Kenya 3829; **FR**: France 00; **ISR**: Israel 98; **TEC**: Tecato (Mexico); **TAB**: Tabasco (Mexico); **IT08**: Italy 08; **IT09**: Italy 09. * Lineage 2. ^+^ Mortality: 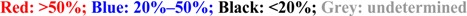. **L**: Low viremia (mean peak viremia ≤ 10^4^ PFU/mL); **M**: Medium viremia (mean peak viremia 10^4^–10^6^ PFU/mL); **H**: High viremia (mean peak viremia > 10^6^ PFU/mL); **na**: Data not available. AFR: Africa; AM: America; AS: Asia; EUR: Europe; OCE: Oceania.

## 4. Pathogenesis

Most of the information currently available about the pathogenesis of WNV infection is derived from experimental studies done in mammals, mostly rodents. The exact mechanism and sites of WNV replication in avian hosts are still not well understood, although with recent experimental infections carried out in a growing number of wild bird species, a great deal has been learned about pathogenesis and antigen distribution [53,91,92,99].

The development of WNV clinical disease in birds is caused by the invasion of major organs such as the liver, spleen, kidney, heart and CNS. In most cases, non-specific clinical signs (ataxia, anorexia, dehydration, *etc.*) appear on days five and six post-infection. Microscopic lesions are often non‑specific and inconsistent. They usually appear first in the spleen and then the virus spreads to other organs, inducing lesions, such as vasculitis, alterations in striated muscle tissues (heart and skeletal muscle), nephritis and hepatitis (WNV pathology and tissue tropism has been thoroughly reviewed in [17]). The appearance of lesions in the CNS occurs later, in a time span that depends on the effectiveness of the immune response and, therefore, the level of viremia.

In highly susceptible species, such as corvids, large amounts of virus are widely distributed in major organs, causing multi-organ failure and inducing a rapid death that does not allow the development of clinical signs [89,92,93]. In such cases, microscopic lesions can be absent in the CNS, while in other organs, pathological changes are acute with minimal inflammatory reaction [91].

In contrast, in birds in which the course of infection is more prolonged, such as some species of raptors and owls, clinical acute disease is infrequent, suffering only mild lesions and low mortality rates. In these hosts, lesions affecting the CNS can be found [66], although antigen immunolabeling is not always possible [54].

Finally, a third clinical picture exists, in which the virus maintains low level of replication that can lead to chronic infections. In some cases, WNV infection can become persistent and it is possible to detect the virus in tissues (mainly spleen, kidney, eye, brain, and skin) several months after initial infection, as it has been demonstrated for House finch (*Haemorhous mexicanus*), House sparrow and Western scrub-jay (*Aphelocoma californica*) surviving both natural and experimental infection [28,67]. Epidemiological consequences of WNV persistence in birds are still not clear [104] but it might play an important role in viral overwintering and mosquito infection in case of host immune impairment and viremia recrudescence (for more details about viral persistence, read subsection 6.3).

Frequently, antigen detection by immunohistochemistry does not correlate well with microscopic lesions or with the viral load detected by real time reverse transcription polymerase chain reaction (real time RT-PCR). One possible explanation would be that pathological changes are induced by the host inflammatory response rather than by direct effect of viral replication [99]. Consequently, lesion description, viral load and antigen detection should be considered together for accurate interpretation of WNV pathogenesis [17].

As aforementioned, considerable differences exist among species in clinical disease pattern as well as in the severity of lesions and antigen distribution. Likewise, important differences in mortality rates are found among orders and even families of birds. As shown in Table 1, viremia levels generally correlate well with mortality rates, being those species that reach the highest viremia titres the ones that usually succumb to the infection (as in the case of most corvid species). However, there are some exceptions, such as American robin (*Turdus migratorius*), Northern cardinal, House sparrow and some species of raptors and owls, that develop high viremia titres but nevertheless, few or none succumb to the infection [5,54,66,79]. Some of these species have been recognized as “super spreaders” with a pivotal role in WNV amplification cycles in the US, based on mosquito feeding preferences, species abundance, high viremia levels, and low mortality rates [62,105].

It is important to highlight that experimental infections cannot completely reproduce field situations. In the wild, numerous contributing factors (such as secondary infections, climate factors, food limitations, *etc.*) exist that cannot be mimicked under laboratory conditions but that have an impact on the distribution and severity of lesions and eventually in the disease outcome. Therefore, is very likely that individuals that survive experimental infections but suffer clinical illness would probably die in the field as a result of difficulties with feeding and/or escaping from predators [43]. Considering this situation, and to assess properly the pathogenicity of a given strain in an experimental setting, it would be of interest to accurately evaluate not only mortality rates but also morbidity indexes (body weight loss, blood biochemistry and hematology alterations, behavioral changes, *etc.*) that would potentially affect survival in the wild [66,92].

## 5. Immune Response

### 5.1. Duration of Humoral Immunity

The observations made in experimental infections of domestic chickens (*Gallus gallus domesticus*) [106] and a large number of wild bird species have indicated that the rise of antibodies against WNV occurs between five and 10 days post-infection (p.i). Antibody levels begin to increase when viremia titres have decreased and the symptoms—if any—are manifest. Due to the limitations in holding wild birds in captivity in BSL3 facilities for long periods, in most cases, birds that survive the infection are euthanized between two to four weeks p.i. or, exceptionally, after nine weeks [5]. Therefore, the record of humoral immunity data is not extended beyond this period. Nevertheless, some studies have been specifically designed to determine the duration of antibodies for longer periods of time. For example, House sparrows and House finches experimentally infected with WNV have been tested at different times p.i. [28,76,104,107], confirming that antibodies remain detectable for at least 28 weeks in House finch [76] and three years in House sparrow, providing sterilizing immunity throughout all this period [107]. Antibody titres reach maximum levels in House sparrow between five and nine weeks p.i. [28]. In Columbiformes, the presence of neutralizing antibodies has been confirmed for at least nine weeks p.i. in Rock pigeon (*Columba livia*) [5] and 30 weeks p.i. in Eurasian collared-dove (*Streptopelia decaocto*) [29]. Studies addressing the time-course of antibody subtypes after WNV infection in avian hosts are scarce. An experiment performed in Rufous night-heron (*Nycticorax caledonicus*) and Little egret (*Egretta garzetta*) showed that neutralizing and haemagglutinin-inhibiting (HI) antibodies rose rapidly from seven to ten days p.i., reaching maximum titres between 10 and 20 days p.i. and steadily declining thereafter up to minimum levels 60–120 days later. In this study, HI antibodies were detected up to 2.5 years p.i. Analysis of Ig subtypes revealed that at 6–7 days p.i. more than 90% of HI antibodies are IgM, declining rapidly so that at day 27 p.i. HI antibodies are almost undetectable. In contrast, IgG levels are very low at six to seven days p.i and rapidly increase reaching a maximum one month after infection [23].

Maternal passive transfer of antibodies has been studied in WNV inoculated domestic chickens [108] showing that all egg yolks and one-day-old chicks from seropositive hens produced neutralizing antibodies that were detectable for at least two weeks post-hatch. Although in most cases antibodies could not be detected at 28 days post-hatch, protection against WNV infection at 42 days post-hatch was observed in some chicks. Nevertheless, in House sparrow, although all seropositive females produced antibody-positive egg yolks, only 20% of them resulted in seropositive chicks [80]. Furthermore, antibodies in these chicks were only detectable the first nine days post-hatch. Consequently, maternal antibodies failed to induce protection in 21–25 days-old chicks, indicating that these antibodies confer protection only during the first days post-hatch. More studies are needed to assess the extent of maternal immunity in other avian species including non-passerine birds, in which a higher persistence of maternal passively inherited antibodies has been observed [109,110,111].

### 5.2. Effect of Previous Exposure to WNV or Other Flaviviruses (Cross-Protection)

Flavivirus cross-protective immunity in birds is considered an important factor to understand transmission ecology in areas where multiple flaviviruses co-circulate [1]. In fact, WNV co-circulates with Saint Louis encephalitis virus (SLEV) in the Americas, Murray Valley encephalitis virus (MVEV) in Australia, Japanese encephalitis virus (JEV) in Southern Asia and Oceania, Usutu virus (USUV) in Africa and Europe and Bagaza virus (BAGV) in Africa, Southern Europe, and India [112,113,114]. One of the hypotheses proposed to explain the lower pathogenicity of WNV in Europe as compared to America points to the past exposure of the reservoir hosts to a wider range of flaviviruses, which might confer immune-mediated cross-protection to WNV [1].

Assessment of cross-protection between different flaviviruses belonging to the same serocomplex usually requires heterologous immunization followed by a challenge with a virulent strain. Such studies have been performed in two epidemiologically relevant wild bird species, House finch [87] and Red-winged blackbird (*Agelaius phoeniceus*) [95]. Immunization of House finches with SLEV induced a complete clinical protection after challenge with WNV, but an incomplete virological protection, as viremia was still detectable, although with much lower titres. In contrast, immunization with WNV did produce sterilizing immunity (absence of viremia) against SLEV [87]. In the case of Red‑winged blackbird, immunization with the NY99 strain induced a nearly complete virological protection against JEV [95]. In all cases, the challenge with the virulent strain elicited immune booster effects, with a considerable increase in neutralizing antibodies. If the objective of an experimental infection is the study of primary host response, it is mandatory to analyze the presence of acquired immunity against WNV (or any other related flavivirus that co-circulate in the area where the birds originate) and exclude positive individuals from the experiment.

Cross-protection not only between different flaviviruses, but between different strains of WNV has also been assessed in wild birds. An experiment conducted by Brault *et al.* [26] demonstrated that pre‑infection of American crows with low pathogenic Old World strains (Kenya and Kunjin), induced total protection against the highly pathogenic NY99 strain. This finding supports the hypothesis that lower virulence of WNV in birds observed in certain areas could be a consequence of co-circulation of low-virulence and high-virulence strains [20].

## 6. Transmission

### 6.1. Arthropod-Borne Transmission

The main transmission route of WNV in wild birds is by mosquitoes that previously have ingested blood from an infected animal acting as reservoir (usually another bird). Species of the genus *Culex* are the main vectors, although others species like *Aedes albopictus* are also competent for virus transmission. Vector capacity of mosquitoes has been confirmed by experimental infection of wild birds, using mosquito bite as the inoculation method. For this purpose, mosquitoes can be inoculated with the virus intrathoracically, or by allowing them to feed upon infectious blood, either from a viremic host or through a system of natural or synthetic membranes. It is well known that the presence of active substances in mosquito saliva enhances arbovirus transmission [115]. Nevertheless, the main disadvantage of this type of experimental inoculation route is that the exact delivered dose is difficult to assess. Following this method, experimental inoculation with different *Culex* species has resulted in successful infection of domestic chicken [106] and 29 wild bird species belonging to 19 families from 11 different orders (Table 2) [5,13,66,81,116]. Furthermore, vector competence of *Culex* mosquitoes has been assessed by analyzing the capability of viral transmission to other vertebrates (mice or birds) after blood feeding from an infected host (Table 2) [13,81,116,117]. 

West Nile virus has been isolated not only from mosquitoes, but also from other haematophagous ectoparasites [9] which might indicate a potential role as vectors for at least some of them. Transmission through tick bites has been proven experimentally in domestic birds [118,119]. In wild birds, only two studies have analyzed the WNV transmission ability of vectors other than mosquitoes: Cliff swallow bug (*Oeciacus vicarius*) and Western black-legged tick (*Ixodes pacificus*) were unable to transmit infectious virus to Cliff swallow (*Petrochelidon pyrrhonota*) and Song sparrow (*Melospiza melodia*), respectively (Table 2) [97,120]. Further studies are needed with other vector and host species to elucidate the role of ectoparasites in amplification and transmission of WNV.

**Table 2:** Arthropod-borne transmission in experimental WNV infections in wild birds.

Vector species		Host		Transmission arthropod-bird	Transmission bird-arthropod-vertebrate *	Ref.
		Order	Species			
**Class: Insecta**						
Order: Diptera	*Culex pipiens*	Passeriformes	House sparrow, Hooded crow	Yes	Yes (mouse)	[13]
		Falconiformes	Common kestrel	Yes	Yes (mouse)	[13]
		Pelecaniformes	Buff-backed heron	Yes	Yes (mouse)	[13]
		Columbiformes	Palm dove	Yes	No (mouse)	[13]
	*Culex univittatus*	Passeriformes	House sparrow, Hooded crow	Yes	Yes (mouse)	[13]
		Pelecaniformes	Buff-backed heron	Yes	-	[13]
		Columbiformes	Palm dove	-	Yes (mouse)	[13]
	*Culex antennatus*	Passeriformes	House sparrow	-	Yes (mouse)	[13]
	*Culex tritaeniorhynchus*	Passeriformes	American robin, American crow, Fish crow, Blue jay, Black-billed magpie, House sparrow, Red-winged blackbird, Common grackle, House finch, European starling	Yes	-	[5]
		Falconiformes	American kestrel	Yes	-	[5,66]
		Strigiformes	Great horned owl	Yes	-	[5,66]
		Galliformes	Northern bobwhite, Japanese quail, Ring-necked pheasant	Yes	-	[5]
		Columbiformes	Mourning dove, Rock pigeon	Yes	-	[5]
		Gruiformes	American coot	Yes	-	[5]
		Anseriformes	Canada goose, Mallard	Yes	-	[5]
		Charadriiformes	Killdeer, Ring-billed gull	Yes	-	[5]
		Psittaciformes	Monk parakeet, Budgerigar	Yes	-	[5]
		Piciformes	Northern flicker	Yes	-	[5]
	*Culex quincefasciatus*	Passeriformes	Cape sparrow, Red bishop	-	Yes (mouse)	[117]
		Passeriformes	Hawai`i `amakihi	Yes	Yes, (Hawai`i `amakihi)	[81]
	*Culex tarsalis*	Passeriformes	House finch	-	Yes (House finch)	[116]
Order: Hemiptera	*Oeciacus vicarius*	Passeriformes	Cliff swallow	-	No (Cliff swallow)	[97]
**Class: Arachnida**						
Order: Acari	*Ixodes pacificus*	Passeriformes	Song sparrow	-	No (Song sparrow)	[120]

* In brackets are indicated the vertebrate animals assayed in the “bird-arthropod-vertebrate” transmission trials.

### 6.2. Contact and Oral Transmission

Although vector borne is the main transmission route for WNV, birds can also get infected through direct contact. Experimental trials that include contact birds for the assessment of potential direct transmission have been performed in 24 species of wild birds belonging to 15 families [5,25,33,37,66,68,101,107]. However, this transmission route has only be demonstrated in a few of them, mainly in Corvidae and Laridae families [5,25], which develop high-titre viremia and shed large amounts of virus in oral and cloacal secretions [5,89]. In these studies, the onset of viremia in contact‑exposed birds began once the mosquito-inoculated birds started to shed virus, suggesting that infection in contact birds occur through fecal-oral or oral-oral routes, or probably by skin or feather picking. Direct contact transmission in laboratory experiments has also been described in Common goose [33], chicken [106] and only in one occasion in Red-legged partridge [101]. In this last case, unexpectedly, infection of contact birds was not delayed with respect to syringe-inoculated partridges, with similar levels of viremia at three days p.i in both groups, an observation difficult to explain. Direct contact transmission could play a role in WNV epidemiology in those situations in which wild birds aggregate in high densities, as in breeding colonies, roosting and feeding areas, or stopovers during migration.

Another means of WNV transmission is through the ingestion of infected food or water. Susceptibility to oral WNV infection has been assayed in 18 species representing 14 families and 8 orders [5,66,96,100]. Great horned owl (*Bubo virginianus*), Eastern screech-owl (*Megascops asio*), American crow, Black-billed magpie (*Pica hudsonia*), and American kestrel (*Falco sparverius*) became infected after consuming infected mice or sparrows [5,66,100]. Infection through consumption of contaminated water has been experimentally observed in Common grackle (*Quiscalus quiscula*), House sparrow and American crow. Moreover, ingestion of infected mosquitoes caused infection in House finch [5]. Nevertheless, other insectivorous species failed to become infected after eating WNV inoculated mosquitoes [5,96]. To test the efficacy of the oral route in transmission compared to the parenteral (syringe or mosquito) route, a group of Song sparrows was subcutaneously inoculated with WNV and another group was fed with infected mosquitoes [96]. This experiment showed that the oral route is much less effective than the parenteral inoculation since the Song sparrows did not become infected orally, even after ingesting several mosquitoes that contained similar or more virus than the dose administered by syringe inoculation.

### 6.3. Persistence of Infection

Opportunities of viral transmission can increase by persistent infection, defined as the detection of virus in host tissues after viremia has subsided. A high viral load in organs caused by persistence of infection might likely result in transmission by predation of infected birds, months after mosquito season. This overwintering mechanism was proposed after the recovery of infectious WNV from the brain of a hawk in New York in February, a period of mosquito inactivity [121]. As explained before for the long-term humoral response evaluation, difficulties in holding WNV-infected wild birds under BSL3 conditions for long periods complicate the determination of viral load in organs of birds a long time after infection. Consequently, few studies have focused on persistence of WNV infection and most of them have been performed in small passerines. In the experiment conducted by Wheeler *et al.* [104], 50% of inoculated House finches and 37% of House sparrows showed persistent infection in spleen and kidney 28 weeks p.i. The virus was still detected by real time RT-PCR in the spleen of two House sparrows at 36 weeks p.i. However, viral isolation attempts were unsuccessful. In a previous work [76], a higher number of organs were analyzed in WNV-infected House sparrows, and viral RNA was detected in juvenile sparrows up to 65 days p.i in kidney and spleen, although infectious virus could be isolated at low titres only in one sparrow at 43 days p.i. The existence of persistent infections was also confirmed in five species of Passeriformes and in Common ground-dove (*Columbina passerina*) by Reisen *et al.* [67]. In this work, the virus was detected in spleen and kidney, but also in lung at >6 weeks p.i and infectious virus was recovered from 4 real time RT-PCR-positive House finches after passage of organ tissue extracts through C6/36 cell culture. All these studies have shown that viral RNA and even infectious virus persists in organs of birds but the mechanisms that allow relapse of viremia levels capable of restarting a transmission cycle have not yet been elucidated.

## 7. Conclusions

The increasing WNV incidence and the raise of new active foci of endemic virus circulation, together with the continuous identification of new strains, make the control of WNV a challenge for animal and public health. Therefore, there is an urgent need to focus research efforts to better understand the transmission dynamics and the virulence determinants of a wide diversity of viral lineages and strains since this knowledge will greatly improve our capacity to control and prevent future outbreaks. For this purpose, experimental infections of reservoir species are of great interest since numerous questions related to the epidemiology and pathogenesis of the disease can only be answered with results of experimental trials. Although the number of WNV strains and wild birds species used in experimental infections has increased considerably in the recent years, there are still many avian hosts and virus strains that need to be tested under laboratory conditions to unravel the peculiar eco-epidemiology found in each affected area. 

Despite logistic constraints derived from the wild origin of birds and the particular conditions of BSL3 facilities, WNV experiments in the natural host are irreplaceable to elucidate the pathologic pathways of the disease, identify main transmission routes and determine host and vector competence. 
